# Gamification in Rehabilitation of Patients With Musculoskeletal Diseases of the Shoulder: Scoping Review

**DOI:** 10.2196/19914

**Published:** 2020-08-25

**Authors:** Bianca Steiner, Lena Elgert, Birgit Saalfeld, Klaus-Hendrik Wolf

**Affiliations:** 1 Peter L. Reichertz Institute for Medical Informatics of TU Braunschweig and Hannover Medical School Braunschweig Germany; 2 Peter L. Reichertz Institute for Medical Informatics of TU Braunschweig and Hannover Medical School Hannover Germany

**Keywords:** shoulder, upper extremity, musculoskeletal diseases, rehabilitation, gamification, serious games, exergames, scoping review

## Abstract

**Background:**

Gamification has become increasingly important both in research and in practice. Particularly in long-term care processes, such as rehabilitation, playful concepts are gaining in importance to increase motivation and adherence. In addition to neurological diseases, this also affects the treatment of patients with musculoskeletal diseases such as shoulder disorders. Although it would be important to assist patients during more than one rehabilitation phase, it is hypothesized that existing systems only support a single phase. It is also unclear which game design elements are currently used in this context and how they are combined to achieve optimal positive effects on motivation.

**Objective:**

This scoping review aims to identify and analyze information and communication technologies that use game design elements to support the rehabilitation processes of patients with musculoskeletal diseases of the shoulder. The state of the art with regard to fields of application, game design elements, and motivation concepts will be determined.

**Methods:**

We conducted a scoping review to identify relevant application systems. The search was performed in 3 literature databases: PubMed, IEEE Xplore, and Scopus. Following the PICO (population, intervention, comparison, outcome) framework, keywords and Medical Subject Headings for shoulder, rehabilitation, and gamification were derived to define a suitable search term. Two independent reviewers, a physical therapist and a medical informatician, completed the search as specified by the search strategy. There was no restriction on year of publication. Data synthesis was done by deductive-inductive coding based on qualitative content analysis.

**Results:**

A total of 1994 articles were screened; 31 articles in English, published between 2006 and 2019, were included. Within, 27 application systems that support patients with musculoskeletal diseases of the shoulder in exercising, usually at home but also in inpatient or outpatient rehabilitation clinics, were described. Only 2 application systems carried out monitoring of adherence. Almost all were based on in-house developed software. The most frequently used game components were points, tasks, and avatars. More complex game components, such as collections and teams, were rarely used. When selecting game components, patient-specific characteristics, such as age and gender, were only considered in 2 application systems. Most were described as motivating, though an evaluation of motivational effects was usually not conducted.

**Conclusions:**

There are only a few application systems supporting patients with musculoskeletal diseases of the shoulder in rehabilitation by using game design elements. Almost all application systems are exergames for supporting self-exercising. Application systems for multiple rehabilitation phases seem to be nonexistent. It is also evident that only a few complex game design elements are used. Patient-specific characteristic are generally neglected when selecting and implementing game components. Consequently, a holistic approach to enhance adherence to rehabilitation is required supporting patients during the entire rehabilitation process by providing motivational game design elements based on patient-specific characteristics.

## Introduction

### Background

Musculoskeletal diseases are one of the leading causes of chronic joint pain and physical disability worldwide [1]. In Germany, the prevalence of chronic pain is approximately 17%, depending on the affected joint [2]. Thereby, chronic shoulder pain belongs to the most common forms of musculoskeletal diseases, which lead to high socioeconomic costs [2]. The most common causes for chronic shoulder pain are shoulder lesions, for example frozen shoulder (6%), osteoarthritis (5%-10%) and rotator cuff tears (10%) [3]. In addition to intense shoulder pain, affected persons also suffer from disabilities in shoulder mobility and functionality [4]. Apart from pain-reducing drugs and surgical treatment, rehabilitative procedures are part of the standard therapy [5]. Rehabilitation is a multifaceted long-term process ranging from inpatient or outpatient orthopedic rehabilitation up to subsequent rehabilitation services [6]. Thereby, the conservative treatment includes a multitude of interventions, such as physical therapy, exercise therapy, psychological treatment, naturopathy, alternative medicine, as well as activities such as swimming or yoga [7,8]. To maintain or improve the success of therapy in patients with musculoskeletal diseases of the shoulder, an ongoing execution of acquired changes in behavior and lifestyle, as well as a long‑term provision of subsequent rehabilitation services is required [9]. Accordingly, patients’ motivation and adherence are crucial factors for effective rehabilitation [10]. However, only 50% of all patients suffering from chronic diseases achieve good adherence [11]. Although therapeutic adherence is defined as “the extent to which a person’s behavior *–* taking medication, following a diet, or executing lifestyle changes, corresponds with agreed recommendations from health care provider [11],” it is not only about following recommendations of physicians or therapists but rather about following general measures to achieve individual therapeutic goals [12]. A multitude of factors influence a patient’s adherence, both in a positive and a negative way [13].

Today’s situation is characterized by a high number of patients with musculoskeletal diseases of the shoulder, low adherence to long-term therapies, and a resulting enormous financial burden on health care systems. This motivates finding modern solutions for supporting patients during rehabilitation and thus reducing costs. Among other things, health-enabling technologies are being developed, which are defined as “sensor-based information and communication technologies, aiming at contributing to a person’s health and health care as well as to his or her quality of life [14].” An increasing number of such health-enabling technologies are supplemented by playful concepts. Particularly in long-term processes, gamification is gaining in importance to increase intrinsic motivation and adherence [15], whereby various game design elements, meaning game components, game dynamics, and game mechanics, are used to generate playfulness [16]. Although it would be important to assist patients during more than one rehabilitation phase, it is hypothesized that existing application systems only support a single phase. It is also unclear which game design elements are currently used in this context and how they are combined to achieve optimal positive effects on motivation and adherence.

### Objectives

Within this scoping review, information and communication technologies utilizing game design elements will be identified to obtain an overview of the fields of application, the game design elements, and motivation concepts. Accordingly, 3 categories should be analyzed: *rehabilitation process*, *gamification*, and *motivation*. As part of these, this review attempts to answer the following questions:

1. Rehabilitation Process

- Scope: What is the scope of the identified application systems?
- Phase: In which phase of rehabilitation processes are the identified application systems used?

2. Gamification

- General implementation: How is the concept of gamification generally implemented in the application systems?
- Game design elements: Which game design elements, that is game components, mechanics, and dynamics, are used?

3. Motivation

- Motivation concepts: Which motivation concepts are addressed?
- Evaluation and outcome: Which aspects of the application systems are evaluated, in particular, motivation and adherence?

## Methods

### Overview

The overall aim of this review was to identify and analyze existing technology-based approaches using game design elements to support rehabilitation processes of patients with musculoskeletal diseases of the shoulder. For this purpose, this scoping review was conducted in accordance with the extended PRISMA checklist for scoping reviews (PRISMA-ScR) [17]. Given the exploratory research question, a scoping review seemed to be more appropriate and thus more effective than a systematic review to get an overview of the field of application and derive appropriate hypotheses.

### Eligibility Criteria

In general, peer-reviewed articles, reviews, and conference articles published between 1997 and mid 2019 were included. For literature not written in English or German, a translator was involved. If, despite all efforts, it was not possible to translate a relevant publication, it was excluded retrospectively. Reviews were only used additionally as part of a citation pearl growing.

The eligibility criteria were determined on basis of the PICO (population, intervention, comparison, outcome) framework [18]; however, *outcome* and *comparison* were not considered. In terms of *population*, all publications focusing on patients with musculoskeletal diseases of the shoulder joint were included. This covered patients with following conditions: fractures, dislocations, inflammatory diseases, and degenerative diseases [19]. Due to treatment features, musculoskeletal diseases such as tumors and tumor-like lesions, congenital diseases of the skeletal system, as well as diseases of the child’s skeletal system were not considered. In terms of *intervention*, all publications describing an application system used to support patients within the rehabilitation process were included. It should be noted that only application systems in which game design elements were used, that is, game components, game mechanics, and game dynamics, were included. Hardware-based systems such as robots, exoskeletons, and orthoses were not considered.

### Information Sources

For a comprehensive overview, we searched 3 databases: the medical literature database Medline via PubMed, the technical research database IEEE Xplore, and the multidisciplinary database Scopus.

### Search Strategy

A proper search term was defined iteratively following PICO. According to the objectives, the key elements *population* (population, patient, problem) and *intervention* were crucial for this review. Patients with musculoskeletal diseases of the shoulder are described by various terms (keywords and Medical Subject Headings) for the shoulder or upper extremities (eg, shoulder, arm, upper limb, or glenohumeral). There was no specific search for musculoskeletal diseases of the shoulder, as it was assumed that these are rarely mentioned explicitly. Intervention describes a variety of different interventions, all of which are subsumed under the term *rehabilitation*. Rehabilitation ranges from diagnosis and acute treatment to therapy planning, medical, or vocational rehabilitation to subsequent rehabilitation services. Therefore, apart from general terms such as *rehabilitation*, *computer*, or *technology-assisted therapy*, more specific therapeutic terms were searched (eg, *training*, *physical therapy*, or *occupational therapy*). Additional terms, such as *therapy planning* or *self-management*, were added to allow a more comprehensive search. Technical aspects were considered by a fourth search block including both specific terms such as *game*, *gamification*, and *exergame*, as well as unspecific terms such as *virtual* and *augmented reality*. For more details on the search queries see Multimedia Appendix 1.

### Synthesis of Results

Following qualitative content analysis [20], full texts classified as relevant were summarized, analyzed, and evaluated. To answer the predefined questions adequately, the basic process was adapted by using the toolbox for qualitative content analysis based on Schreier [21]. Here, an essential part was the deductive-inductive approach for creating a category system. The starting point was a code tree consisting of 16 deductively built subcategories and 6 main categories: (1) target group, (2) field of application, (3) rehabilitation phase, (4) motivation aspects, (5) game characteristics, and (6) evaluation. The applied methodology of mixed deductive-inductive categorization allowed a quantitative evaluation in addition to a purely qualitative analysis of publications [20]. In order to simplify and partially automate the analysis, qualitative data analysis software MAXQDA (version 2018.2; VERBI GmbH) was used.

### Selection, Categorization, and Data Extraction

**Figure 1:**
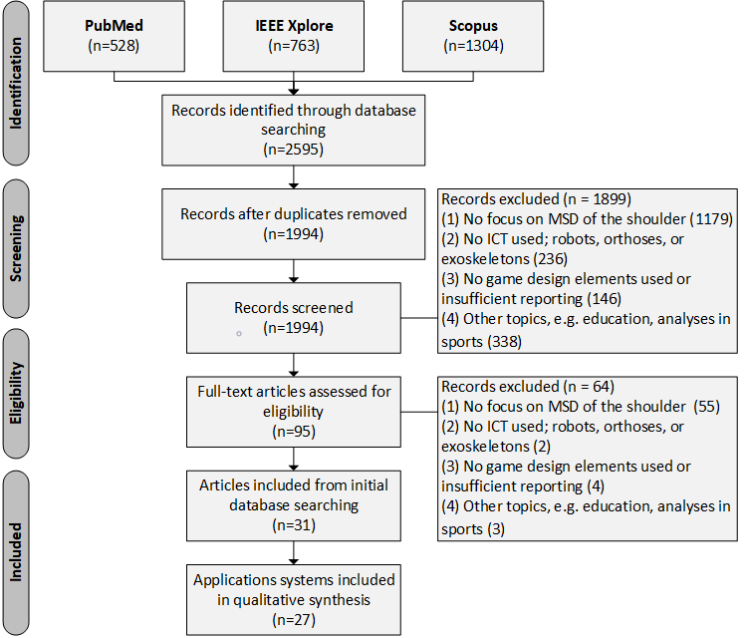
PRISMA Flow diagram for the identification of application systems. ICT: information communication technology; MSD: musculoskeletal diseases.

The search request was made to PubMed on June 17, 2019 and to IEEE Xplore and Scopus on June 18, 2019. Overall, the search returned 2595 matches. After excluding duplicates, title screening and abstract screening of the remaining 1994 publications were conducted by 2 independent reviewers (LE, BSt) with different expertise—physical therapy and medical informatics. Checking of predefined exclusion criteria was done sequentially. Whenever disagreements arose, a third reviewer (BSa) was consulted for decision making. Thus, a total of 95 publications were included in the full-text screening. Full-text screening was carried out in the same way as the title and abstract screening. Finally, 31 publications were included in the full-text analysis (Figure 1).

Qualitative content analysis was performed by a single reviewer (BSt) using the predefined search tree. During the analysis, 17 inductive subcategories were added (see Multimedia Appendix 2 for more details).

## Results

### Overview

A total of 31 articles published between 2006 and 2019 were selected for full-text analysis. These articles introduced 27 different application systems to support the rehabilitation of patients with musculoskeletal diseases of the shoulder by using game design elements. A list of identified articles grouped by the underlying population can be found in Multimedia Appendix 3, and a complete overview of all analyzed aspects can be found in Multimedia Appendix 4.

### Population

Of the 27 application systems, 10 had been developed explicitly for a musculoskeletal disease of the shoulder—6 for frozen shoulder, 4 for shoulder impingement syndrome; 2 focused on different musculoskeletal diseases of the shoulder similar in physiotherapeutic treatment (eg, rotator cuff tear and humerus fracture); 11 others outlined the target group only vaguely—there were 5 application systems for shoulder injuries, 2 for musculoskeletal shoulder pain, and 4 for musculoskeletal diseases of upper extremities, in general; and the remaining 4 were intended to be used for treatment of musculoskeletal diseases of the shoulder as well as of one of the following diseases: paraplegias with spinal cord injuries [22], cerebrovascular diseases [23], arm amputations [24], or elbow and radius fractures [25]. Here, it seems questionable whether a combined approach for musculoskeletal and neurological diseases is appropriate for the individual therapeutic needs of a patient. While patients with such neurological diseases may also suffer from pain and limited range of motion of upper extremities [26-28], the causes and symptoms, the patients’ self-management skills, and the therapeutic goals may differ. Especially for severely affected patients, there are nuances in the therapy plans, exercise programs, and ultimately, also in the physiotherapeutic exercises to be performed.

### Rehabilitation Process

#### Scope

All 27 application systems assist in the execution of exercises, mostly physiotherapeutic self-exercises. Of them, 5 were explicitly described as telerehabilitation systems [25,29-32]. These application systems provide additional functionalities for creating and adjusting individual exercises and training plans or for monitoring training progress by a therapist or physician; however, only Rahman et al [33] and Luchessi et al [30] describe possibilities for monitoring patients’ adherence. The application system GEAR, for example, has a dashboard for physicians to monitor adherence by visualizing the exercise performance in different diagrams [29,33]. Beyond this, the app SHOULPHY claimed to offer possibilities for disease management [30]. Given the existing definitions of disease management [34,35], this does not fully apply. Although patient empowerment is increased and physicians are supported in the development and maintenance of a treatment plan [34], there is no holistic, cross-process view on care over the entire life cycle of the disease [35].

#### Phase

**Figure 2:**
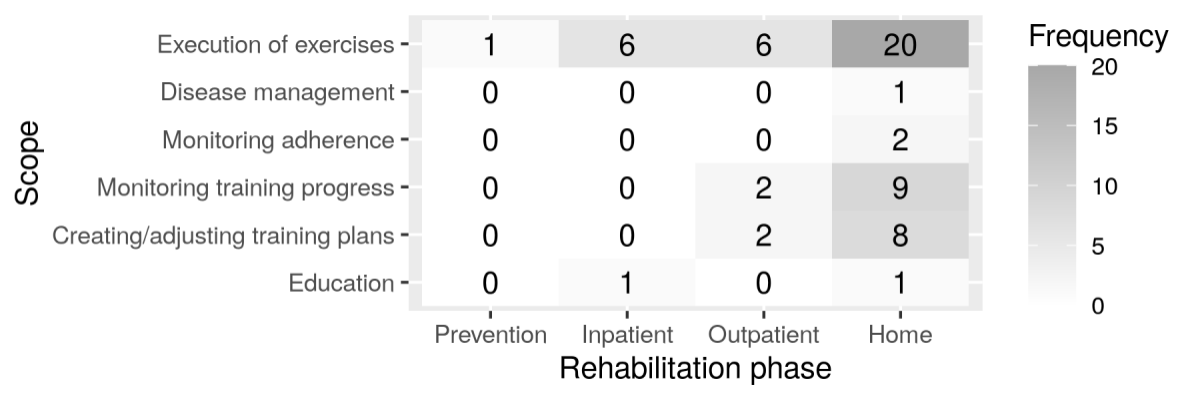
Heatmap of frequencies of scopes in individual rehabilitation phases; outpatient and inpatient refer to a rehabilitation facility (multiple mentions are possible in both axes).

Most of the application systems were developed for the outpatient sector as part of conservative therapy or subsequent rehabilitation services (Figure 2). Only Mangal et al [36] addressed the additional employment of a Kinect-based rehabilitation system for treatment of frozen shoulder in inpatient rehabilitation. Vogt et al [37] and Wiederhold et al [24] add 2 training systems explicitly developed for inpatient rehabilitation. PhysioSonic, for example, is a sonification system for shoulder patients installed at an orthopedic hospital [37]. Besides the multitude of training systems for later rehabilitation phases, Huang et al [38] are the only ones presenting an app for prevention. Using a bicycle ergometer, Google Earth, and a Kinect camera, exercises based on pilates can be performed.

### Gamification

#### General Implementation

Of the 27 application systems, 24 were based on self-developed software products. Only 3 made use of commercial games, namely Wii Sports [39,40]; EyeToy: Play, Dance Dance Revolution; and Taiko Drum Master [24]. While commercial games do not require any investments for development, in contrast to in-house developed games, they are not specially adapted to the target group. In terms of the selection and implementation of exercises and game design elements, it would be necessary to include patient-specific characteristics. However, only 2 consider either of these characteristics, that is, age and gender [41,42].

Whereas 11 publications focus on the implementation of a single, sometimes incredibly broad game [22,23,29,37,38,43-48], just as many publications provide several games [24,28,32,36,39-42,49-51]. By implementing different games, a higher variability is gained. Thus, motivation and acceptance of users can be additionally increased by avoiding boredom. Furthermore, it is possible to match therapeutic interventions much better to the patients’ state of health. A user’s personal preferences can be also addressed, even if only indirectly. For example, in the application system InMotion users can choose to touch butterflies in a beautiful garden, hit and shoot paintballs towards a canvas, control a rowboat through several checkpoints, or mimic the motions of a pendulum [42].

#### Game Components

**Figure 3:**
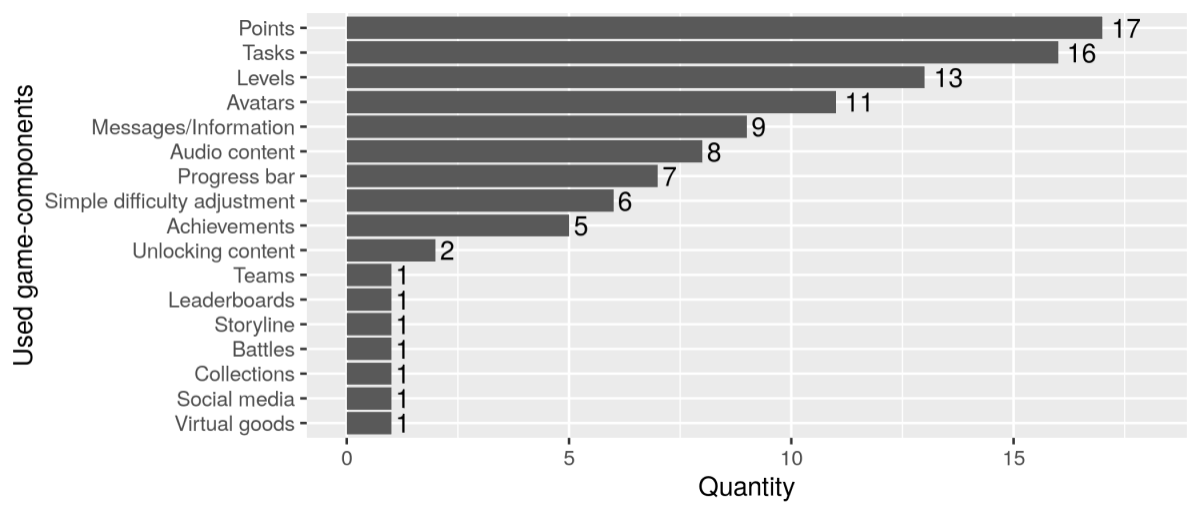
Overview of game components ordered by frequency.

The most frequently used game components are those that are easy to implement, such as points, tasks, avatars, and information messages (Figure 3). When playing Cupid’s Arrow, for example, players collect points by shooting an arrow at a heart through correct arm movements [41]. Also, in Classic Clock, points can be earned by imitating movements of a pendulum as precisely as possible [42]. However, points can also be used in application systems implementing no specific game. Da Gama et al [52] demonstrate how points can be gained by completing physiotherapeutic exercises correctly, thus boosting the total score of a therapy session.

Most application systems implement training as playful tasks. Apart from being more fun, this is more interesting than doing the same monotonous exercises on a daily basis [44]. In addition to simple tasks such as picking fruits [25,47,50,53], placing dishes [50‑51], or fishing [44], there are more complex, and therefore, possibly more exciting and less fatiguing tasks. Examples are destroying submarines by cleverly dropping bombs [41] and controlling objects (eg, aircrafts, storks, rays) along a course with or without obstacles [23,25,32,38]. Probably the most extensive game in terms of implemented tasks and game components is The Sorcerer’s Apprentice [45]. Here, a mage (avatar) must be controlled in a virtual world (storyline). Artefacts must be collected (tasks, virtual goods) to unlock and execute different shoulder exercises (unlocking content). Successfully completed exercises are rewarded with items and bonus points (collection, points).

#### Game Mechanics

**Figure 4:**
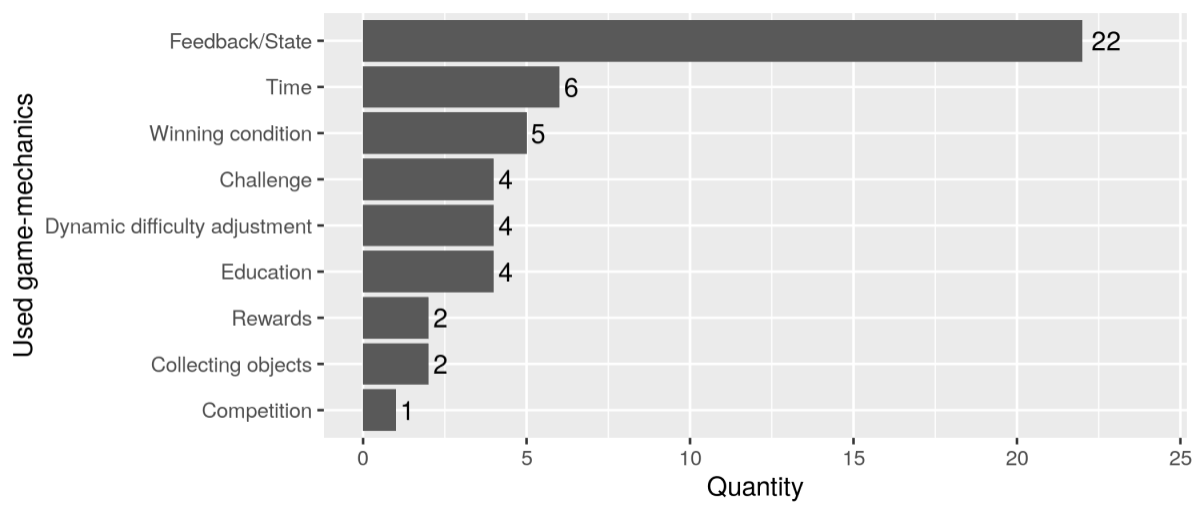
Overview of game mechanics ordered by frequency.

Nearly all application systems used game components, such as points, progress bars, leaderboards, and achievements to provide feedback or represent a current state (Figure 4). Since all application systems were implemented to support training, half do not give feedback on the game (game feedback) but instead give feedback on the performance of exercises (therapy feedback). Therapy feedback often quantifies either the quality of exercise performance [37,42,45,46,52] or the progress/current status [44,50] to motivate patients or to ensure correct practice. Almost all application systems provided instructions, or even complete tutorials, for exercising correctly. Nevertheless, there were few providing additional information or education about rehabilitation. Only the exergame by Nava et al [48] made information on the relevance of continuous training available. More complex game mechanics were rarely implemented. Cooperation, round-based games, transaction, and coincidence were not found at all.

#### Game Dynamics

According to García-Martínez et al [54], all implemented tasks are based on the same 6 game dynamics (activities) for therapeutic games: (1) follow the path, (2) touch the target, (3) point and shoot the target, (4) free movement, (5) move the target, and (6) catch the target. Most commonly used are follow the path (12 of the 27) and touch the target (11 of the 27), as this is closest to normal, conscious movements of exercise therapy for the shoulder. Activities such as free movement, move the target, and catch the target, on the other hand, are used less frequently, as they can lead more quickly to incorrect movements and thus injuries or overstraining [55]. Quite often, a combination of different movements, and thus game dynamics, takes place to motivate users and to respond to different therapy needs.

### Motivation

#### Motivation Concepts

Encouraging motivation, as well as enhancing engagement, is one of several goals of gamification [56,57]. Certain publications describe their application systems as motivating (6 of the 27), without mentioning specific factors for this effect [23,25,32,36,39,46]. Furthermore, motivational effects are often based on general, superficial aspects, such as interactivity (12 of the 27), fun, enjoyment, and entertainment (13 of the 27). In some cases, there are also more concrete aspects mentioned, such as engagement (11 of the 27) and avoiding boredom (10 of the 27). Just 4 publications addressed the need for a better understanding of the exercises to be performed, in terms of the therapeutic goals [37,44] and the associated faster learning of exercises [42,53]. Considering established motivation models, for instance the self-determination theory of motivation [58], it becomes clear how these aspects represent an elementary factor for intrinsic motivation.

#### Evaluation and Outcome

Except for 3 publications presenting purely application systems or approaches [23,47,59], all application systems were evaluated on at least one aspect. Frequently, this was the evaluation of technical feasibility (10 of the 27) and usability (9 of the 27) by means of pilot studies or simple informal pretests. Resulting from the use of gamification, motivation is another frequently analyzed criterion (13 of the 27). This is described either as intrinsic motivation, motivation to perform exercises, or motivation to regularly perform therapy sessions. Of 14 application systems, 5 were rated as motivating in pretests or pilot studies [22,29,36,48,52]. However, it is unclear to what extent these results are transferable to the population owing to small sample sizes [29,52], healthy people as users [29,36,48,52], or informal testing [22]. The remaining application systems simply suppose an increase in motivation (9 of the 27)—motivating effects were usually deduced from single subjective questions [36-38,42] or directly assumed without any evidence as a result of the game design elements used [24,30,40,41,45]. The same applied to adherence analyzes; 3 publications presume an increase was caused by the game design elements that had been implemented, although there was no evidence given [29,30,40].

## Discussion

### Principal Findings

A previous review [60] has already shown the high number of application systems available for assisting patients with neurological disorders during rehabilitation, varying from Parkinson disease, stroke, and cerebral palsy. For rehabilitation of musculoskeletal diseases, in contrast, only a few publications can be found. This scoping review identified a total of 27 application systems to support patients with musculoskeletal diseases of the shoulder during their rehabilitation by using game design elements. It is remarkable that only about one-third of these application systems were designed for a specific musculoskeletal disease of the shoulder. Musculoskeletal diseases of the shoulder, such as shoulder dislocation or humeral head fracture, are not given special consideration.

The few application systems that can be found are mostly exergames supporting physiotherapeutic self-exercises, usually at home (20 of the 27). Application systems to assist prevention, diagnostics, acute treatment, or inpatient rehabilitation seems to be almost nonexistent. The same applies to application systems supporting multiple rehabilitation phases. Here, not even one could be discovered.

There is also little variation in the scope of application systems. All application systems support the training of patients, but only a few can be used to create training plans (8 of the 27), or even, to adjust them during the rehabilitation process (6 of the 27). Also, functionalities for monitoring therapy adherence and progress are quite rare (9 of the 27). Just 2 offer possibilities for monitoring therapy adherence, although adherence is a crucial factor for sustainable rehabilitation success [10], whereby an adequate management of the underlying musculoskeletal diseases is necessary to achieve adherence throughout the entire lifecycle of the disease. Luchessi et al [30] are the only ones who actually mention the term *disease management*.

Almost all application systems are based on in-house developed software (24 of the 27). They offer flexibility in terms of the therapeutic needs of patients with shoulder diseases as well as of the game design elements used to enhance motivation and adherence. Selection and implementation of adequate game design elements depend substantially on the target group and its individual characteristics, such as age, gender, personal preferences, level of knowledge, and intrinsic motivation [15,42]. However, the identified application systems generally describe the user group exclusively by indication for treatment. Only 2 approaches address gender and age differences. Other patient-specific characteristics have not yet been considered in selecting nor in implementing game design elements.

Analyzing the application systems in terms of the game design elements used, it is obvious that more complex game components are used only rarely or not at all. Usually, easy to implement game components are selected and combined. Since the realization of game mechanics is based on the game components implemented, even here, little variation is found. Accordingly, 22 application systems used diverse simple game components to provide feedback. Astonishingly, education was also rarely included, although this is a meaningful aspect for patient empowerment. Overall, it seems that only little attention has been paid to the factual effect of individual game components and game mechanics when selecting and implementing them. No publications deal with respective motivation models and theories to demonstrate or prove the effects of single game design elements on user behavior.

Almost all application systems had been evaluated. Mostly, this concerned the technical feasibility, efficacy, or acceptance but rarely the motivation and adherence of patients. Factual evidence of increased motivation or adherence caused by the developed application systems or game design elements as part of clinical trials was lacking.

### Limitations

There were only a few application systems for the rehabilitation of patients with musculoskeletal diseases of the shoulder using game design elements. Excluding hardware-based systems limited the search considerably. The same applied to the exclusion of publications due to insufficient descriptions of game design elements during title and abstract screening. Here, numerous publications were excluded (n=150). Given an inadequate description of game design elements in the abstract, it is not possible to determine for certain that such elements had not been implemented. However, this indicated that gamification does not have a central role in these publications. For analyses with regard to the motivational effects of game design elements, this is nevertheless, an essential prerequisite.

Whereas all screenings were performed by 2 independent reviewers, the qualitative content analysis was done by a single reviewer. A second reviewer might have ensured the correct identification and classification of relevant game design elements, rehabilitation phases, and motivation aspects. Particularly in classifying game mechanics and game dynamics, there is some margin for interpretation. Consequently, it was not possible to provide details on game dynamics in the sense of conceptual structures, such as constraints, emotions, or relationships [61]. However, to avoid incorrect classifications, in case of hesitation, one of the other reviewers was always consulted.

Overall, it is striking that only exergames supporting outpatient or inpatient rehabilitation could be identified. There was just one application system for prevention and one for disease management. This restricted result might be caused by the common meaning of rehabilitation as medical and vocational rehabilitation including subsequent rehabilitation services. Even searching for terms such as *self-management*, *prevention*, and *patient pathway* was not sufficient. Therefore, a complementary mobile health app analysis is planned using the Google Play Store to examine whether there are other application systems beyond exergames and subsequent rehabilitation services for musculoskeletal diseases of the shoulder.

### Conclusions

The concept of gamification is gaining importance in the context of health care to enhance motivation and support therapy in general. Especially for chronic diseases and thus long-term care processes such as rehabilitation, gamification reveals particular potential. However, for rehabilitation of musculoskeletal diseases of the shoulder, only a few application systems that use game design elements to increase motivation and adherence exist. Beyond that, all identified application systems focus on the support of self-exercises, mainly in the outpatient sector. Application systems for inpatient rehabilitation, other rehabilitation phases, or multiple rehabilitation phases seem to be nonexistent. Only the fewest application systems provide additional functionalities, for example, provision of information or monitoring of adherence to assist patients during rehabilitation and increase their self-management skills. Altogether, the selection, combination, and implementation of game design elements appears somewhat impetuous. Indeed, gamification is generally used to motivate, avoid boredom, and distract from pain and anxiety. But it seems that only a little attention has been paid to the factual effect and thus benefit of individual game components and game mechanics. Furthermore, patient-specific characteristics are mostly neglected when selecting game components. It remains exciting to learn about the effects that future developments will report, including patient-specific game design elements.

## Appendix

Multimedia Appendix 1 Search queries.

Multimedia Appendix 2 Code tree.

Multimedia Appendix 3 Search results grouped by the underlying population.

Multimedia Appendix 4 Complete overview of search results.

## Abbreviations

PICO: population, intervention, comparison, outcome
PRISMA: Preferred Reporting Items for Systematic Reviews and Meta-Analysis
