# Supplementary material for: Gamification in Rehabilitation of Patients With Musculoskeletal Diseases of the Shoulder: Scoping Review
Source: JMIR Serious Games. 2020 Aug 25;8(3):e19914. doi: 10.2196/19914 (PMC7479582; doi:10.2196/19914)
Supplement: Multimedia Appendix 1 [file games_v8i3e19914_app1.pdf]

Search queries

Medline via Pubmed

((„upper extremity“[MeSH Terms] OR „upper extremity“[Title/Abstract] OR „upper limb“[Title/Abstract] OR shoulder[Title/Abstract] OR glenohumeral[Title/Abstract])

AND

(rehabilitation[MeSH Terms] OR rehabilitation[Title/Abstract] OR „exercise therapy”[Title/Abstract] OR „self-management”[Title/Abstract] OR physiotherapy[Title/Abstract] OR „physical therapy“[Title/Abstract] OR exercise[MeSH Terms] OR exercise*[Title/Abstract] OR „pain management”[MeSH Terms] OR „pain management”[Title/Abstract] OR „drug therapy”[MeSH Terms] OR „Hyperthermia, Induced”[MeSH Terms] OR „Social Change”[MeSH Terms] OR „psychological treatment”[Title/Abstract] OR „social work”[MeSH Terms] OR „Patient Care Planning”[MeSH Terms] OR „Patient Care Planning”[Title/Abstract] OR „Preoperative Care”[MeSH Terms] OR „Preoperative Care”[Title/Abstract] OR „Postoperative Care”[MeSH Terms] OR „Postoperative Care”[Title/Abstract] OR „therapy, computer-assisted“[MeSH Terms] OR „computer-assisted therapy“[Title/Abstract] OR „technology-assisted therapy“[Title/Abstract] OR „disease management”[MeSH terms] OR „disease management”[Title/Abstract] OR „patient pathway”[Title/Abstract] OR „treatment pathway”[Title/Abstract] OR „Patient Education as Topic”[MeSH terms] OR „patient education”[Title/Abstract])

AND

(game* OR gami* OR exergam*[Title/Abstract] OR „serious game”[Title/Abstract] OR „video games“[MeSH Terms] OR virtual[Title/Abstract] OR „augmented reality”[Title/Abstract])

NOT

(stroke[Title] OR amputee*[Title] OR „multiple sclerosis“[Title] OR neuroreha*[Title] OR Orthos*[Title] OR paresis[Title] OR parkinson*[Title] OR „phantom limb pain“[Title] OR prosthe*[Title] OR spastic[Title] OR „spinal cord“[Title] OR tetraplegi*[Title]))

Scopus

((TITLE-ABS-KEY("upper extremity") OR TITLE-ABS("upper limb") OR TITLE-ABS(shoulder) OR TITLE-ABS(glenohumeral))

AND

(TITLE-ABS-KEY(rehabilitation) OR TITLE-ABS("exercise therapy") OR TITLE-ABS("self-management") OR TITLE-ABS(physiotherapy) OR TITLE-ABS("physical therapy") OR TITLE-ABS-KEY(exercise*) OR TITLE-ABS-KEY("pain management") OR KEY("drug therapy") OR KEY("Hyperthermia, Induced") OR KEY("Social Change") OR TITLE-ABS("psychological treatment") OR KEY("social work") OR TITLE-ABS-KEY("Patient Care Planning") OR TITLE-ABS-KEY("Preoperative Care") OR TITLE-ABS-KEY("Postoperative Care") OR TITLE-ABS-KEY("computer-assisted therapy") OR TITLE-ABS("technology-assisted therapy") OR TITLE-ABS-KEY("disease management") OR TITLE-ABS("patient pathway") OR TITLE-ABS("treatment pathway") OR KEY("Patient Education as Topic") OR TITLE-ABS-KEY("patient education"))

AND

(game* OR gami* OR TITLE-ABS(exergam*) OR TITLE-ABS("serious game") OR KEY("video game*") OR TITLE-ABS(virtual) OR TITLE-ABS("augmented reality"))

AND NOT

(TITLE(stroke) OR TITLE(amputee*) OR TITLE("multiple sclerosis") OR TITLE(neuroreha*) OR TITLE(Orthos*) OR TITLE(paresis) OR TITLE(parkinson*) OR TITLE("phantom limb pain") OR TITLE(prosthe*) OR TITLE(spastic) OR TITLE("spinal cord") OR TITLE(tetraplegi*))

AND

(LIMIT-TO(DOCTYPE,"ar") OR LIMIT-TO(DOCTYPE,"cp") OR LIMIT-TO(DOCTYPE,"re") OR LIMIT-TO(DOCTYPE,"sh")))

IEEE Xplore

(("Mesh_Terms":"upper extremity" OR "Document Title":"upper extremity" OR "Document Title":"upper limb" OR "Document Title":shoulder OR "Document Title":glenohumeral)

AND

("Mesh_Terms":rehabilitation OR "Document Title":rehabilitation OR "Document Title":"exercise therapy" OR "Document Title":"self-management" OR "Document Title":physiotherapy OR "Document Title":"physical therapy" OR "Mesh_Terms":exercise OR "Document Title":exercise* OR "Mesh_Terms":"pain management" OR "Document Title":"pain management" OR "Mesh_Terms":"drug therapy" OR "Mesh_Terms":"Hyperthermia, Induced" OR "Mesh_Terms":"Social Change" OR "Document Title":"psychological treatment" OR "Mesh_Terms":"social work" OR "Mesh_Terms":"Patient Care Planning" OR "Document Title":"Patient Care Planning" OR "Mesh_Terms":"Preoperative Care" OR "Document Title":"Preoperative Care" OR "Mesh_Terms":"Postoperative Care" OR "Document Title":"Postoperative Care" OR "Document Title":"computer-assisted therapy" OR "Document Title":"technology-assisted therapy" OR "Mesh_Terms":"disease management" OR "Document Title":"disease management" OR "Document Title":"patient pathway" OR "Document Title":"treatment pathway" OR "Mesh_Terms":"Patient Education as Topic" OR "Document Title":"patient education")

AND

(game* OR gami* OR "Document Title":exergam* OR "Document Title":"serious game" OR "Mesh_Terms":"video games" OR "Document Title":virtual OR "Document Title":"augmented reality"))

(("Mesh_Terms":"upper extremity" OR "Abstract":"upper extremity" OR "Abstract":"upper limb" OR "Abstract":shoulder OR "Abstract":glenohumeral)

AND

("Mesh_Terms":rehabilitation OR "Abstract":rehabilitation OR "Abstract":"exercise therapy" OR "Abstract":"self-management" OR "Abstract":physiotherapy OR "Abstract":"physical therapy" OR "Mesh_Terms":exercise OR "Abstract":exercise* OR "Mesh_Terms":"pain management" OR "Abstract":"pain management" OR "Mesh_Terms":"drug therapy" OR "Mesh_Terms":"Hyperthermia, Induced" OR "Mesh_Terms":"Social Change" OR "Abstract":"psychological treatment" OR "Mesh_Terms":"social work" OR "Mesh_Terms":"Patient Care Planning" OR "Abstract":"Patient Care Planning" OR "Mesh_Terms":"Preoperative Care" OR "Abstract":"Preoperative Care" OR "Mesh_Terms":"Postoperative Care" OR "Abstract":"Postoperative Care" OR "Abstract":"computer-assisted therapy" OR "Abstract":"technology-assisted therapy" OR "Mesh_Terms":"disease management" OR "Abstract":"disease management" OR "Abstract":"patient pathway" OR "Abstract":"treatment pathway" OR "Mesh_Terms":"Patient Education as Topic" OR "Abstract":"patient education")

AND

(game* OR gami* OR "Abstract":exergam* OR "Abstract":"serious game" OR "Mesh_Terms":"video games" OR "Abstract":virtual OR "Abstract":"augmented reality"))

(("Mesh_Terms":"upper extremity" OR "Index Terms":"upper extremity" OR "Index Terms":"upper limb" OR "Index Terms":shoulder OR "Index Terms":glenohumeral)

AND

("Mesh_Terms":rehabilitation OR "Index Terms":rehabilitation OR "Index Terms":"exercise therapy" OR "Index Terms":"self-management" OR "Index Terms":physiotherapy OR "Index Terms":"physical therapy" OR "Mesh_Terms":exercise OR exercise* OR "Mesh_Terms":"pain management" OR "Index Terms":"pain management" OR "Mesh_Terms":"drug therapy" OR "Mesh_Terms":"Hyperthermia, Induced" OR "Mesh_Terms":"Social Change" OR "Index Terms":"psychological treatment" OR "Mesh_Terms":"social work" OR "Mesh_Terms":"Patient Care Planning" OR "Index Terms":"Patient Care Planning" OR "Mesh_Terms":"Preoperative Care" OR "Index Terms":"Preoperative Care" OR "Mesh_Terms":"Postoperative Care" OR "Index Terms":"Postoperative Care" OR "Index Terms":"computer-assisted therapy" OR "Index Terms":"technology-assisted therapy" OR "Mesh_Terms":"disease management" OR "Index Terms":"disease management" OR "Index Terms":"patient pathway" OR "Index Terms":"treatment pathway" OR "Mesh_Terms":"Patient Education as Topic" OR "Index Terms":"patient education")

AND

(game* OR gami* OR "Index Terms":exergam* OR "Index Terms":"serious game" OR "Mesh_Terms":"video games" OR "Index Terms":virtual OR "Index Terms":"augmented reality"))
