# Supplementary figures and images for: Gamification in Rehabilitation of Patients With Musculoskeletal Diseases of the Shoulder: Scoping Review
Source: JMIR Serious Games. 2020 Aug 25;8(3):e19914. doi: 10.2196/19914 (PMC7479582; doi:10.2196/19914)

# Code tree

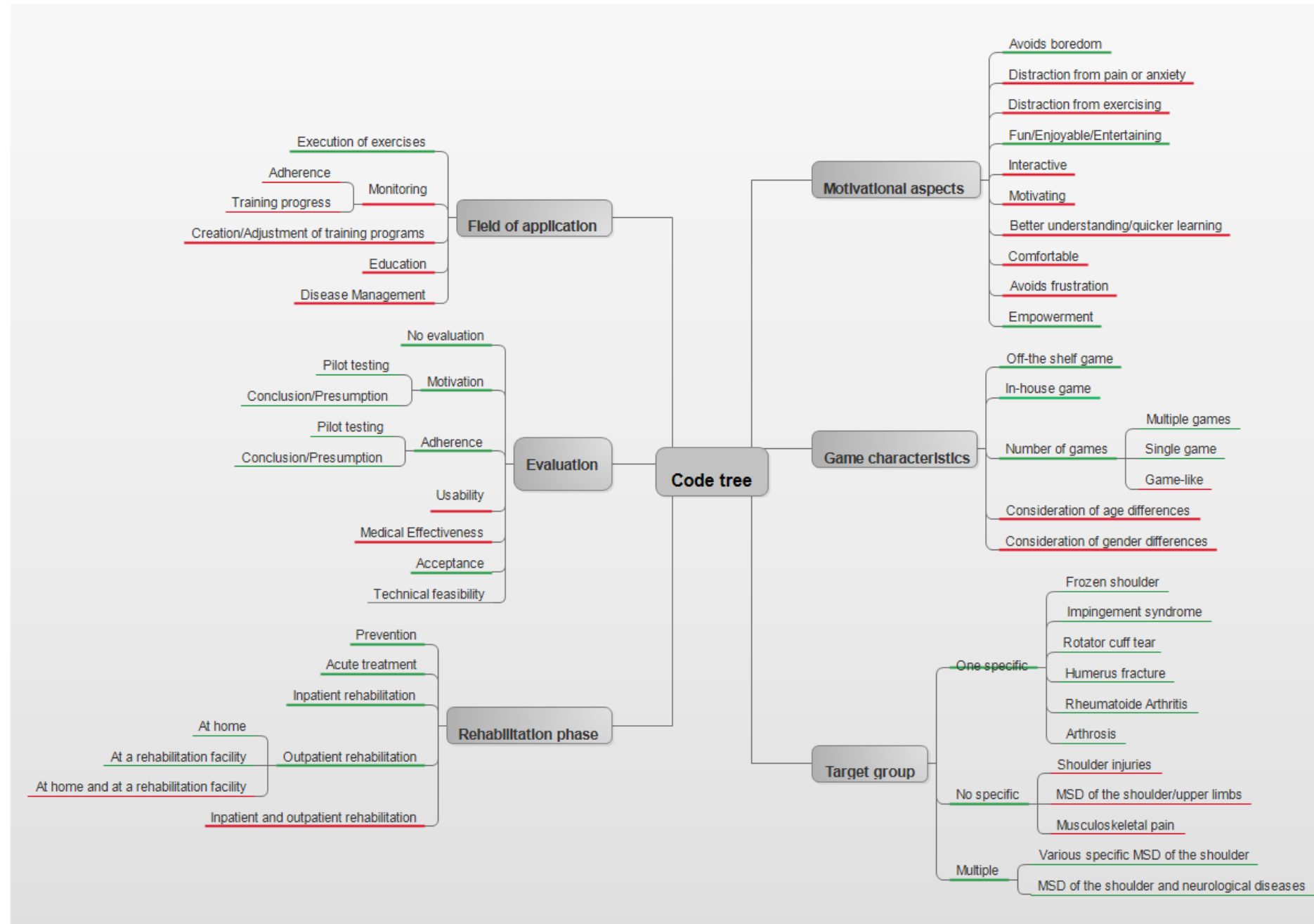

Supplement: Multimedia Appendix 2 [file games_v8i3e19914_app2.pdf]
