# Supplementary material for: Gamification in Rehabilitation of Patients With Musculoskeletal Diseases of the Shoulder: Scoping Review
Source: JMIR Serious Games. 2020 Aug 25;8(3):e19914. doi: 10.2196/19914 (PMC7479582; doi:10.2196/19914)
Supplement: Multimedia Appendix 3 [file games_v8i3e19914_app3.pdf]

Search results grouped by the underlying population

Titles of articles	First author, year	Application
**Frozen Shoulder**		
An Interactive Kinect-Based Game Development for Shoulder Injury Rehabilitation	Arif A, 2018	unstated
The App Game Interface Design for Frozen Shoulder Rehabilitation	Chung CE, 2017	MoMo
An IMU-compensated Skeletal Tracking System Using Kinect for the Upper Limb	Du YC, 2018	unstated
Intelligent Frozen Shoulder Rehabilitation	Huang MC, 2014	unstated[*1]
The Development of Interactive Shoulder Joint Rehabilitation System Using Virtual Reality in Association with Motion-sensing Technology	Yeh SC, 2014	unstated[*1]
Frozen Shoulder Rehabilitation Using Microsoft Kinect	Mangal NK, 2017	unstated
GEAR: A Mobile Game-Assisted Rehabilitation System	Rahman M, 2016	GEAR[*2]
GEAR Analytics: A Clinician Dashboard for a Mobile Game Assisted Rehabilitation System	Rahman M, 2016	GEAR[*2]
**Shoulder Impingement Syndrome**		
The Sorcerer's Apprentice A Serious Game Aiding Rehabilitation in the Context of Subacromial Impingement Syndrome	Fikar P, 2013	The Sorcerer's Apprentice
Comparison of Virtual Reality Exergaming and Home Exercise Programs in Patients with Subacromial Impingement Syndrome and Scapular Dyskinesis: Short Term Effect	Pekyavas NO, 2017	commercial (Wii Sports)
Daily Life Self-management and Self-treatment of Musculoskeletal Disorders Through SHOULPHY	Lucchesi I, 2018	SHOULPHY
Structured Wii Protocol for Rehabilitation of Shoulder Impingement Syndrome: A Pilot Study	Rizzo JR, 2017	commercial (Wii sports)
**Shoulder Injuries**		
Serious Rehabilitation Games with Kinect	Fernandez-Cervantes V, 2015	unstated
Guidance and Movement Correction Based on Therapeutics Movements for Motor Rehabilitation Support Systems	Da Gama A, 2012	unstated
Therapy-led Design of Home-Based Virtual Rehabilitation	Powell V, 2015	unstated
PhysioSonic - Evaluated Movement Sonification as Auditory Feedback in Physiotherapy	Vogt K, 2010	PhysioSonic
A VR-based User Interface for the Upper Limb Rehabilitation	Shi Y, 2018	unstated
**Musculoskeletal Shoulder Pain**		
The Design of a Shoulder Rehabilitation Game System	Chang CM, 2010	unstated [*3]
An Interactive Game-based Shoulder Wheel System for Rehabilitation	Chang CM, 2012	unstated [*3]
Designing an Exergaming System for Exercise Bikes Using Kinect Sensors and Google Earth	Huang SY, 2017	unstated
**Musculoskeletal Diseases of the Shoulder/Upper Limbs in General**		
Usability Study of a Kinect-Based Rehabilitation Tool for the Upper Limbs	Muñoz GF, 2019	KineActiv R
Prototype of a Shoulder and Elbow Occupational Health Care Exergame	Nava W, 2015	unstated

Development and Assessment of a Physiotherapy System Based on Serious Games	Symeonidis I, 2016	PARC
Adaptive Gameplay and Difficulty Adjustment in a Gamified Upper-Limb Rehabilitation	Pinto JF, 2018	InMotion
**Several Specific Musculoskeletal Diseases of the Shoulder**		
Comparing Outcomes of Kinect Videogame-Based Occupational/Physical Therapy Versus Usual Care	Dahl-Popolizio S, 2014	unstated
Assistance System for Rehabilitation and Valuation of Motor Skills	Quevedo WX, 2017	unstated
**Musculoskeletal Diseases and Others**		
Alpha Testing of the Rapid Recovery Kayaking-based Exergame	Shewaga R, 2015	Rapid Recovery*4
Rapid Recovery: A Kayaking-based Exergame for Shoulder Rehabilitation and Physical Fitness	Shroeder B, 2014	Rapid Recovery*4
A Wearable Rehabilitation Game Controller Using IMU Sensor	Yin ZX, 2018	unstated
Evaluation of Virtual Reality Therapy in Augmenting the Physical and Cognitive Rehabilitation of War Veterans	Wiederhold BK, 2006	commercial
A Low Cost Virtual Reality System for Rehabilitation of Upper Limb	Budziszewski P, 2013	Unstated

(* = articles describing the same application)
